# Diagnostic and prognostic significance of ALU-based cell-free DNA in colorectal cancer: a systematic review and meta-analysis

**DOI:** 10.3389/fonc.2024.1398062

**Published:** 2024-08-07

**Authors:** Mohammad Taghizadeh-Teymorloei, Leila Alizadeh, Somaieh Matin, Tohid Jafari-Koshki, Abbas Karimi

**Affiliations:** ^1^ Department of Molecular Medicine, Faculty of Advanced Medical Sciences, Tabriz University of Medical Sciences, Tabriz, Iran; ^2^ Gastroenterology and Liver Diseases Research Center, Tabriz University of Medical Sciences, Tabriz, Iran; ^3^ Department of Internal Medicine, School of Medicine, Ardabil University of Medical Sciences, Ardabil, Iran; ^4^ Molecular Medicine Research Center (MMRC), Department of Statistics and Epidemiology, Faculty of Health, Tabriz University of Medical Sciences, Tabriz, Iran; ^5^ Department of Epidemiology and Biostatistics, School of Medicine, Urmia University of Medical Sciences, Urmia, Iran

**Keywords:** ALU, colorectal cancer, diagnosis, prognosis, sensitivity, specificity

## Abstract

**Introduction:**

Colorectal cancer (CRC) is a major global health concern. This study aimed to investigate the role of ALU-based cell-free DNA (cfDNA) in the diagnosis and prognosis of CRC.

**Methods:**

We selected relevant literature from PubMed, Scopus, Web of Science, EMBASE, and Science Direct databases based on strict inclusion and exclusion criteria. 17 eligible studies were included in the final analysis (13 studies for diagnostic and 4 studies for prognostic meta-analysis). The search covered relevant publications up to July 1, 2024.

**Results:**

The pooled sensitivity, specificity, and diagnostic odds ratios (DOR) of ALU-based cfDNA in CRC diagnosis were 0.81 (95% CI= [0.70, 0.89]), 0.90 (95% CI= [0.70, 0.96]), and 40.58 (95% CI= [17.87, 92.19]), respectively. The area under the ROC curve was 0.92 (95% CI= [0.89, 0.94]). Patients with higher concentrations of plasma/serum ALU-based cfDNA had poorer overall survival (OS) (pooled hazard ratio = 2.33 ([95% CI= [1.80, 3.03]).

**Conclusion:**

The current evidence supports the utility of circulating ALU as a promising non-invasive diagnostic and prognostic tool for CRC. Furthermore, as a potential biomarker, ALU-based cfDNA could play a significant role in clinical application.

**Clinical implications:**

The evidence suggests that circulating ALU-based cell-free DNA (cfDNA) holds promise as a non-invasive diagnostic and prognostic tool for colorectal cancer, potentially enhancing clinical decision-making.

**Systematic review registration:**

https://www.crd.york.ac.uk/prospero/, identifier PROSPERO (CRD42023486369).

## Introduction

Colorectal cancer (CRC) is a severe disease that poses a substantial threat to human health. According to the 2024 global cancer data (GLOBCAN 2024) given by the International Agency for Research on Cancer (IARC), the incidence of CRC is listed as the third most frequent, with around 935,173 people dying from CRC each year globally, making it the second biggest cause of cancer-related fatalities (1–3). The 5-year survival rate for CRC decreases significantly from 95% at stage I to just 6% at stage IV, indicating a strong correlation between the stage at diagnosis and the death rate. Early detection of CRC is critical to improving survival because it reduces the chance of death, stops the disease from spreading, and enhances the quality of life and long-term health outcomes (4). While fecal occult blood tests and colonoscopies are standard diagnostic tools, their invasive nature and the discomfort they cause often lead to patient aversion and subsequent diagnostic delays (5, 6). The search for dependable biomarkers has resulted in the use of blood-based markers like carcinoembryonic antigen (CEA) and carbohydrate antigens (CA 19–9 and CA 242) (7), and alpha-fetoprotein (AFP) (8); however, their diagnostic efficacy is marred by low sensitivity and specificity, particularly in benign conditions. This limitation necessitates the exploration of novel biomarkers that can offer more precise diagnostic and prognostic insights for high-risk CRC patients (9–12). Thus, searching for new biomarkers in CRC diagnosis and prognosis has attracted many researchers. There is considerable interest in non-invasive liquid biopsy (LB) as a supplementary and maybe alternative approach, attempting to address these limitations (13–16). LB is a process that extracts tumor-derived biomarkers from body fluids such as blood, urine, saliva, feces, or cerebrospinal fluid. This approach may identify a number of circulating tumor components, including circulating tumor cells (CTCs), circulating tumor DNA (ctDNA), messenger RNA (mRNA), non-coding RNA, extracellular vesicles (EVs), and tumor-educated platelets (TEPs). LB is essentially a non-invasive diagnostic tool that aids in the early diagnosis and monitoring of cancer (17, 18).

Recent studies emphasize circulating cell-free DNA (cfDNA) analysis as a possible biomarker for many therapeutic applications in cancer, such as diagnosis, evaluation of tumor development, assessment of therapy response, and prognosis (19–21). cfDNA includes DNA that enters the bloodstream from the breakdown of cells or is actively secreted by normal and cancerous tissues. It contains important genetic details about the primary tumor’s structure. When collecting tumor biopsy is difficult, analyzing cfDNA in Blood can serve as an alternative for histopathological examination (22, 23). Furthermore, the amount of cfDNA is typically changed in malignancies, even in the early stages (24). Higher levels of blood cfDNA are reported to have a strong association with malignancy and poorer clinical outcomes. Over the last two decades, cfDNA analysis has become a promising technique for cancer diagnosis and prognosis (25, 26).

Arthrobacter luteus (ALU) fragments are primate-specific repeats that make up approximately 11% of the human genome (27). The ALU fragments are short interspersed nuclear elements (SINEs) that are common in the human genome. They are transposable elements that may be affected by environmental factors (28) and ALUs have wide-ranging influences on gene expression, affecting polyadenylation, splicing, and ADAR editing (27). They significantly influence the composition of cfDNA (26). These fragments are widely utilized as target molecules in LB applications due to their abundant presence throughout the human genome. Analyses focused on Alu-based cfDNA have demonstrated promising application potential in several key areas of cancer management (29): Numerous studies have employed major repetitive regions within the human genome, specifically ALU115 and ALU247, for quantifying cell-free DNA (cfDNA). ALU115 reflects total DNA content, encompassing both longer and shorter cfDNA fragments. In contrast, ALU247 specifically identifies tumor-derived DNA, focusing on longer cfDNA fragments associated with malignancy (30, 31). ALU115/247 has been intensively researched as a promising non-invasive diagnostic and prognostic marker in the area of LB, with a wide variety of quantitative and qualitative results (32, 33).

Thus, searching for new biomarkers in CRC diagnosis and prognosis has attracted many researchers. The present study aimed to carry out the first meta-analysis to quantitatively analyze the diagnostic and prognostic accuracy of ALU-based cfDNA in CRC patients.

## Materials and methods

**Figure 1:**
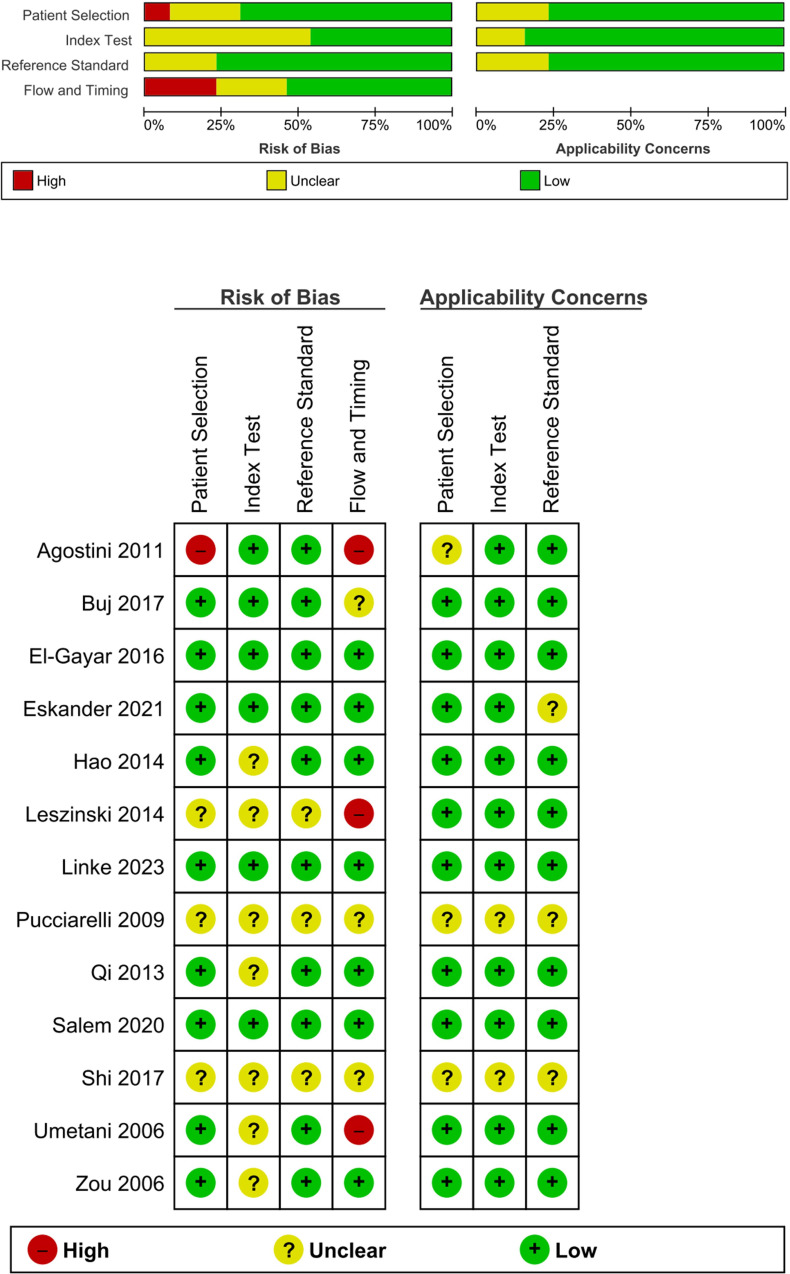
Assessing risk of bias and applicability concerns based on the QUADAS-2 tool.

This report has been organized following the PRISMA 2020 framework, as depicted in **Figure 1** (34), additionally, we have incorporated the PRISMA 2020 Checklist (35) in **Supplementary Table 1** to ensure comprehensive reporting and methodological transparency.

### Search strategy

The proposed protocol has been filed in the PROSPERO (CRD) International Prospective Registry of Systematic Reviews with the identification number CRD42023486369. We searched PubMed, Embase, ScienceDirect, Scopus, and the Web of Science Citation Index for research on the diagnostic or prognostic use of ALU-based cfDNA for CRC before July 1, 2024, without specifying a start date. Initially, no language limitations were in place. Our search approach was as follows. For diagnosis: (cancer OR Malignancy OR neoplasm OR tumor OR carcinoma OR “adenomatosis” OR “polyposis” OR APCL) AND (colon OR rectal OR colorectal) AND (“cell-free DNA” OR “Cell-Free Nucleic Acids” OR “circulating DNA” OR cfDNA) AND (ALU OR “Arthrobacter luteus”) AND (diagnoses OR “sensitivity and specificity” OR “sensitivity” OR “ specificity” OR “ROC curve” OR “area under curve” OR “AUC”).

For prognosis: (cancer OR Malignancy OR neoplasm OR tumor OR carcinoma OR “adenomatosis” OR “polyposis” OR APCL) AND (colon OR rectal OR colorectal) AND (“cell-free DNA” OR “Cell Free Nucleic Acids” OR “circulating DNA” OR cfDNA) AND (ALU OR “Arthrobacter luteus”) AND (Prognosis OR “Survival Analysis” AND “Overall Survival (OS)” OR “Disease-Free Survival (DFS)” OR “Progression-Free Survival (PFS)” OR “Recurrence/Relapse-free survival (RFS)” OR “Minimal Residual Disease (MRD)” OR “Kaplan–Meier Estimate”). Moreover, the search for other related terms included “Relapse-Free Survival”, “Locoregional Relapse-Free Survival”, “Distant Metastasis Free Survival (DMFS)”, “Liquid Biopsy Analysis”, “Prognostic Value”, “Prognostic Factor”, “Prognostic Indicator”, “Prognostic and Predictive Biomarkers”, “Recurrence Risk”, “Predictive for Outcome”.

To ensure completeness, we checked the reference lists of all included papers and discovered another relevant study. No attempts were made to recover unpublished studies.

### Eligibility criteria

The main criteria for study inclusion were: (1) reporting research on patients with CRC; (2) detecting ALU in plasma, serum, feces, or tissues; (3) making a definitive diagnosis of CRC with the gold standard; (4) conducting a thorough investigation into the relationship between ALU and clinical endpoints including OS, DFS, cancer-specific survival (CSS), RFS; and (5) providing enough data to calculate true positive (TP), false positive (FP), false negative (FN), and true negative (TN).

Studies were excluded if they were: (1) not relevant to the study topic; (2) published as letters, reviews, editorials, or case reports; (3) duplicate publications; (4) non-English publications; or (5) involved unqualified data. Other unrelated publications, such as ongoing articles, editorials, commentaries, book chapters, and others, were also excluded.

### Data extraction

Two independent reviewers (MT and AK) collected relevant data from the papers using standardized procedures. Any issues about the inclusion of specific research were handled by talking with a third reviewer (TJ) and reaching a consensus. The following information was extracted from the diagnostic and prognostic studies: name of the first author, time of publication, country of research, number of participants, sample source, and diagnostic results including sensitivity, specificity, TP, FP, FN, and TN; or prognostic results including follow-up time and HR estimates with 95% confidence intervals (CIs) for OS.

### Quality assessment

Two reviewers (MT and AK) assessed the quality of the selected diagnostic research publications using the Quality Assessment of Diagnostic Accuracy Studies 2 (QUADAS-2) (36). Both authors independently reviewed the studies and resolved any discrepancies through consensus.

### Risk of bias

To assess bias and applicability, the QUADAS-2 tool from RevMan 5.4 was utilized (37). In the “patient selection” domain, two studies had a high risk of bias, two had an unclear risk, and 10 had a low risk. For the “index test” domain, seven studies had a high risk, two had a low risk, and five had an unclear risk. The “reference test” had a low risk of bias with three studies having an unclear risk, none with a high risk, and 11 with a low risk. In the “flow and timing” domain, 11 studies had a low risk, one had a high risk, and two had an unclear risk. Applicability concern was low, indicating a high degree of applicability due to the preselection of articles. The results are shown in **Figure 1**.

### Statistical analysis

In the conducted meta-analyses, the following statistical methods were employed for analyzing diagnostic and prognostic measures. For the diagnostic meta-analyses, the numbers of patients with TP, FP, FN, and TN test findings were either directly collected or recalculated using provided diagnostic estimates and sample sizes. These data were then evaluated using a bivariate meta-analysis model (38). To assess various diagnostic performance measures such as sensitivity, specificity, positive likelihood ratio (PLR), negative likelihood ratio (NLR), diagnostic odds ratio (DOR), and diagnostic score. Additionally, the summary receiver operator characteristic (SROC) curve and the area under the SROC curve (AUC) were computed, with AUC serving as a metric to evaluate the diagnostic power of the test. Study heterogeneity and publication bias were assessed using the I2 statistic and Deeks’ funnel plot, respectively (39, 40).

In the prognostic meta-analyses, hazard ratios (HRs) and their corresponding 95% confidence intervals (CI) were combined to estimate the overall impact of the variable of interest on the OS of CRC patients. Study heterogeneity was assessed utilizing Cochran’s Q test and the I2 statistic (ranging from 0% to 100%) (41). A random-effects model was used to synthesize the overall effect (42). Finally, Begg’s funnel plot was used to examine the probability of publication bias among the included papers (43). All statistical analyses were performed using the Stata17.0 statistical software program (Stata Corporation, College Station, Texas, USA) and RevMan 5.4 (37). Results with p<0.05 were considered statistically significant.

## Results

### Key finding

ALU-based cfDNA are widely utilized as target molecules in LB applications due to their abundant presence throughout the human genome. Analyses focused on ALU-based cfDNA have demonstrated promising application potential in several key areas of cancer management: This meta-analysis evaluated the diagnostic and prognostic performance of Alu-based cfDNA in CRC. The diagnostic meta-analysis showed that ALU-based cfDNA had a combined sensitivity of 0.81 and a specificity of 0.90, with an area under the summary receiver operating characteristic (SROC) curve of 0.92, indicating good diagnostic accuracy. The prognostic meta-analysis revealed that higher levels of ALU-based cfDNA were associated with shorter OS in CRC patients (HR = 2.33, 95% CI: 1.80–3.03).

### Selection of studies

**Figure 2:**
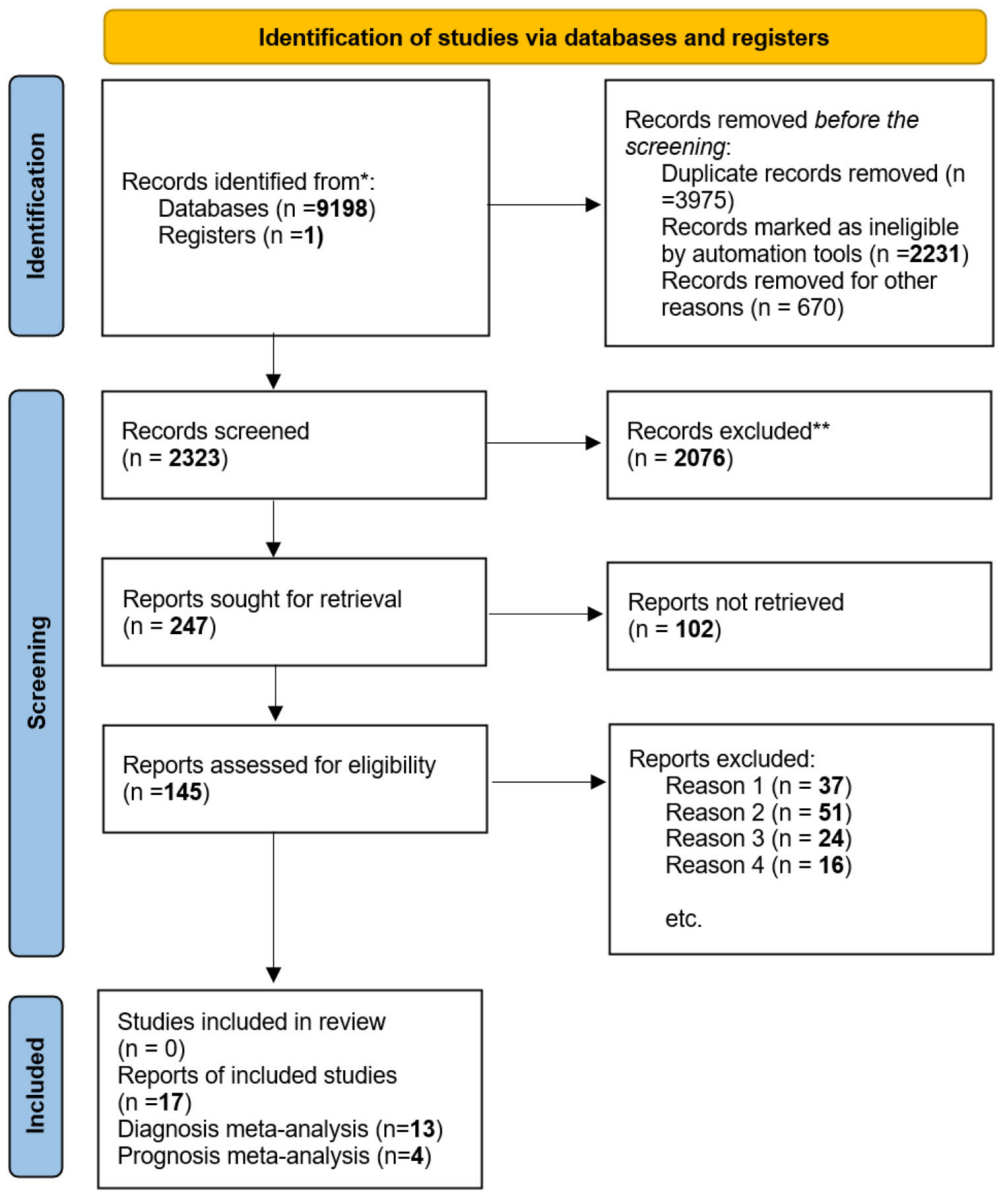
In this study, we utilized the ‘PRISMA 2020 flow diagram’ template, which is a visual representation of the flow of information through the different phases of a systematic review. The template includes searches of only databases and registers. Please refer to the PRISMA flow diagram of the study for more details. * The trial registers including clinicaltrials.gov and trial search.who.int/AdvSearch.aspx were used in our search strategies. ** The titles and abstracts of articles are not relevant to our research questions. Reason 1. Not related to our topic. Reason 2. Not relevant to diagnosis or prognosis. Reason 3. Without sufficient data. Reason 4. Not available data and not about human studies.

**Figure 2** shows the flow diagram for the literature search. The first search of chosen literature databases and other sources returned 9198 items. After a comprehensive exclusion procedure at each stage, 17 published studies were eventually included in this meta-analysis, consisting of 13 studies for diagnostic meta-analysis and 4 studies for prognostic meta-analysis; in terms of prognosis, these four studies were connected with OS.

### Diagnostic meta-analysis

#### Study characteristics and quality assessment

A total of 13 papers fulfilled the inclusion criteria for quantitative analysis of circulating ALU-based cfDNA in CRC detection. These studies comprised 807 patients with CRC and 496 healthy controls. All patients with CRC underwent histopathological investigation. **Table 1** summarizes the general features of this research. The sample sources included serum (n = 4) (30, 31, 52, 53), plasma (n = 6) (45–49, 51), feces (n = 1) (54), Formalin-Fixed Paraffin-Embedded (FFPE) (n = 1) (50), and blood (n = 1) (44). Nine studies evaluated ALU115/247 for diagnostic value (30, 31, 46–49, 51–53), two studies evaluated ALU (50, 54), and two other studies analyzed ALU-based cfDNA (44, 45). Additionally, 12 publications assessed ALU-based cfDNA using q-PCR (30, 31, 45–54), while only one publication analyzed ALU-based cfDNA by Branched DNA (b-DNA) (44). The included studies were conducted in Egypt (n = 4) (30, 48, 52, 53), China (n = 3) (31, 44, 45), USA (n = 2) (47, 54), Germany (n = 2) (49, 51), Italy (n = 1) (46), and Spain (n = 1) (50). With the exception of one study that did not determine the stages of CRC, all studies included all stages of CRC. **Table 1** summarizes the features of these investigations.

**Table 1:** Characteristics of the included studies for the diagnostic value of ALU-based cfDNA in CRC patients.

NO.	First Author	Country	ALU type	Specimen	Tumor stages	Method of detection	No. of C	No. of P	Sens* %	Spec** %	AUC	Ref
1	Hongzhi Zou (2006)	USA	ALU	Stool	I-IV	qPCR	20	18	44	100	–	
2	Jing Qi (2013)	China	ALU	Blood	I-IV	b-DNA	92	31	64.5	98.9	0.904	(44)
3	Hao Shi (2020)	China	ALU	Plasma	I-IV	qPCR	20	71	100	44	0.734	(45)
4	Marco Agostini (2011)	Italy	ALU115/247	Plasma	0-IV	qPCR	35	67	94	100	Alu 115 = 0.92; Alu 247 = 0.97	(46)
5	T B Hao (2014)	China	ALU115/247	Serum	I-IV	qPCR	110	205	69.23	99.09	0.85	(31)
6	T B Hao (2014) (integrity index)***	China	ALU115/247	Serum	I-IV	qPCR	110	205	73.08	97.27	0.89	(31)
7	Naoyuki Umetani (2006)	USA	ALU115/247	plasma	I-IV	qPCR	51	32	41	90	0.75	(47)
8	Nancy Samir Eskander (2021)	Egypt	ALU115/247	plasma	I-IV	qPCR	43	43	73.7	66.7	0.695	(48)
9	Christian Linke (2023)	Germany	ALU115/247	plasma	I-IV	qPCR	50	80	82	67	0.69 - 0.82	(49)
10	Raquel Buj (2016)	Spain	ALU	FFPE	–	qPCR	16	16	80	82	0.926	(50)
11	Gloria leszinski (2014)	Germany	ALU115/247	plasma	–	qPCR	24	24	75	70.8	Alu115 = 0.738; Alu247 = 0.729	(51)
12	Dina El-Gayar (2016)	Egypt	ALU115/247	Serum	II-IV	qPCR	20	50	90	85	0.9	
13	Alhanafy (2017)	Egypt	ALU115	Serum	I-IV	qPCR	20	80	83	90	–	
14	Ramy Salem (2020)	Egypt	ALU115/247	Serum	I-IV	qPCR	30	90	93	65	0.95	

*Sensitivity.

** Specificity. *** Integrity index was calculated as the ratio of ALU-qPCR result (ALU247 and ALU115).

### Diagnostic accuracy of ALU alone in CRC

**Figure 3:**
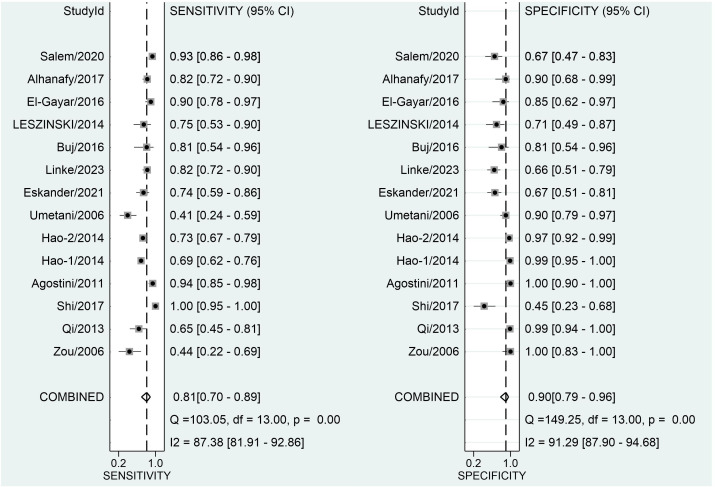
Forest plot displaying the combined sensitivity and specificity of ALU-based cfDNA in diagnosing CRC.

**Figure 4:**
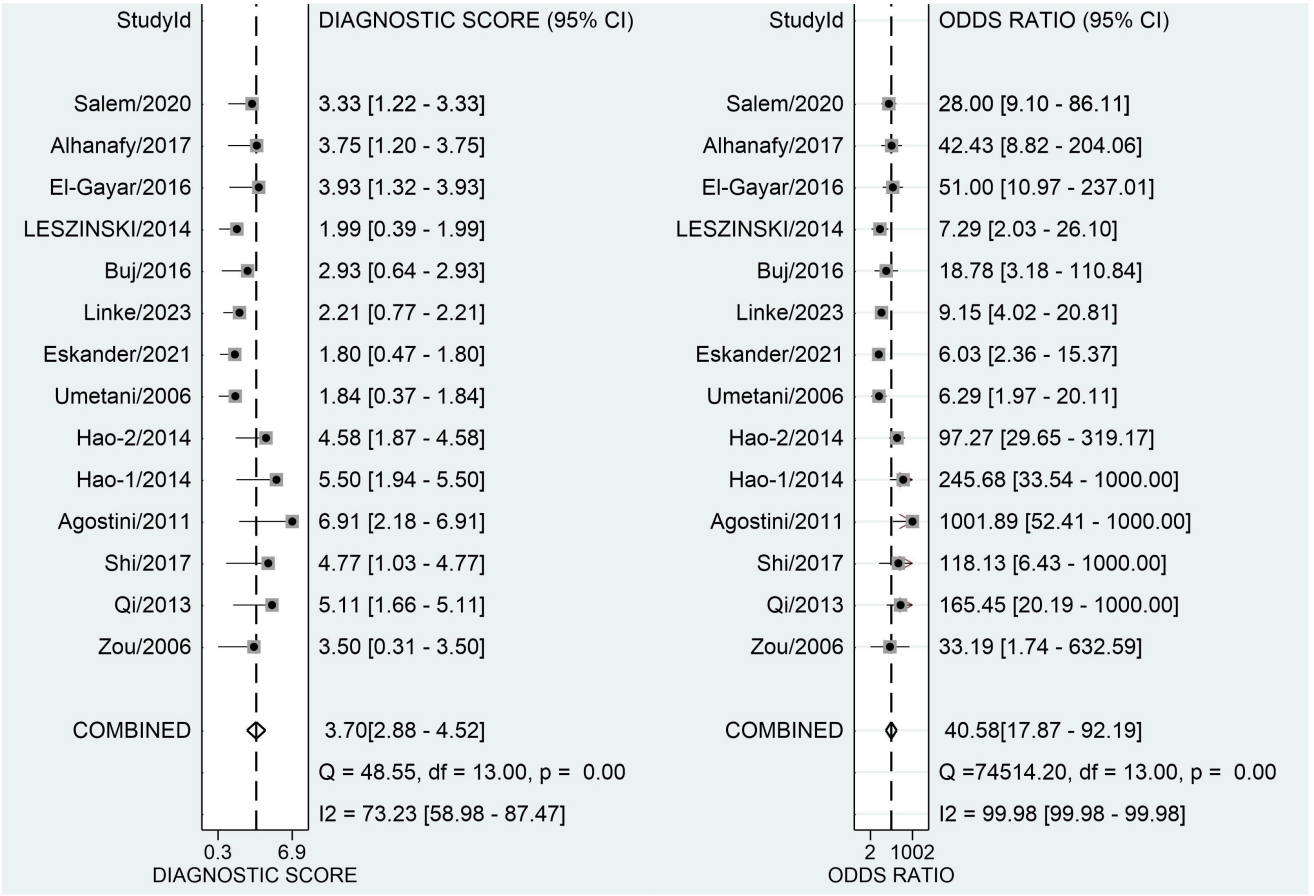
Forest plot displaying the combined diagnostic score and odds ratio of ALU-based cfDNA in diagnosing CRC.

**Figure 5:**
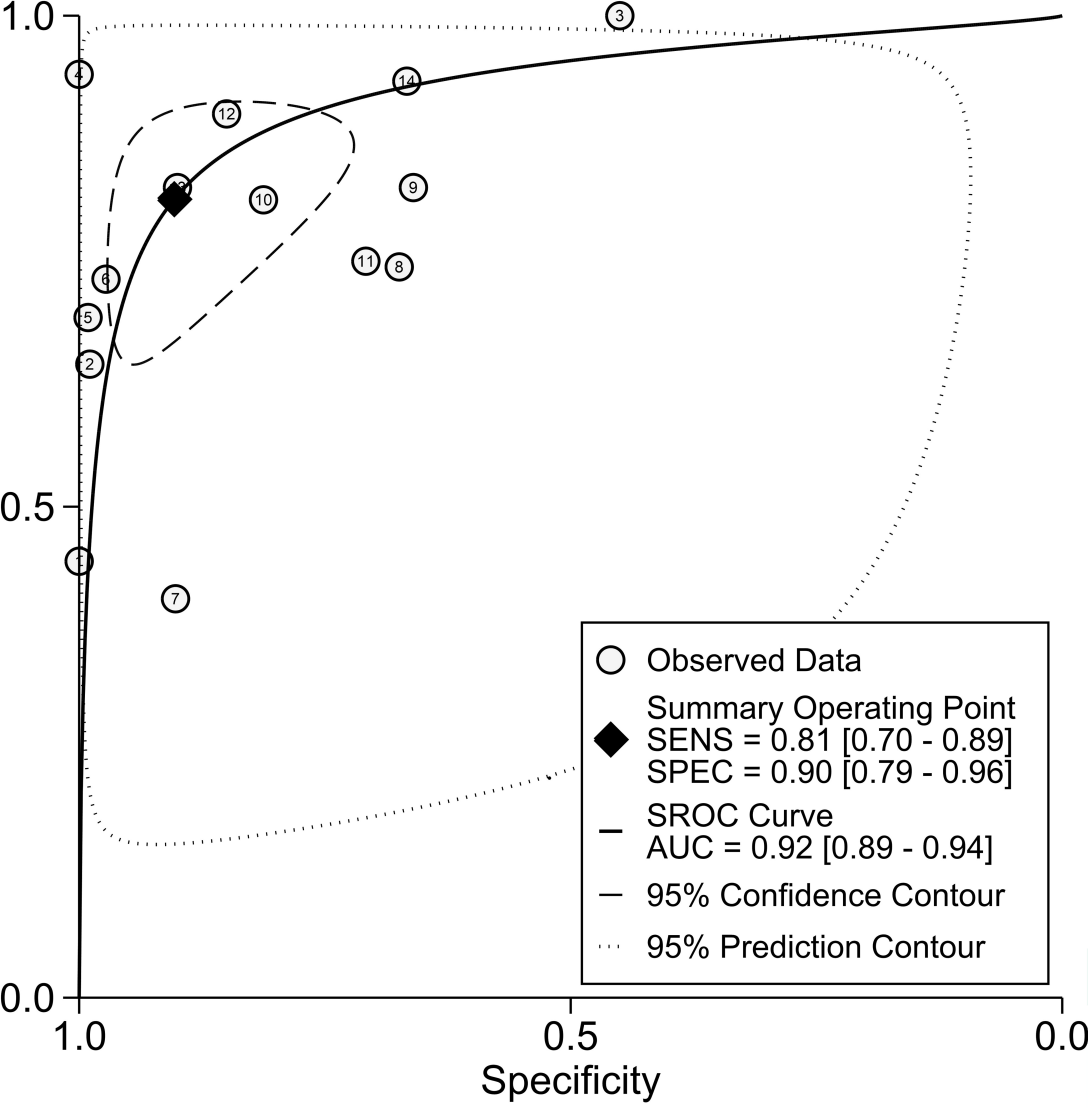
SROC curves in the diagnosis value of ALU-based cfDNA in CRC.

**Figure 6:**
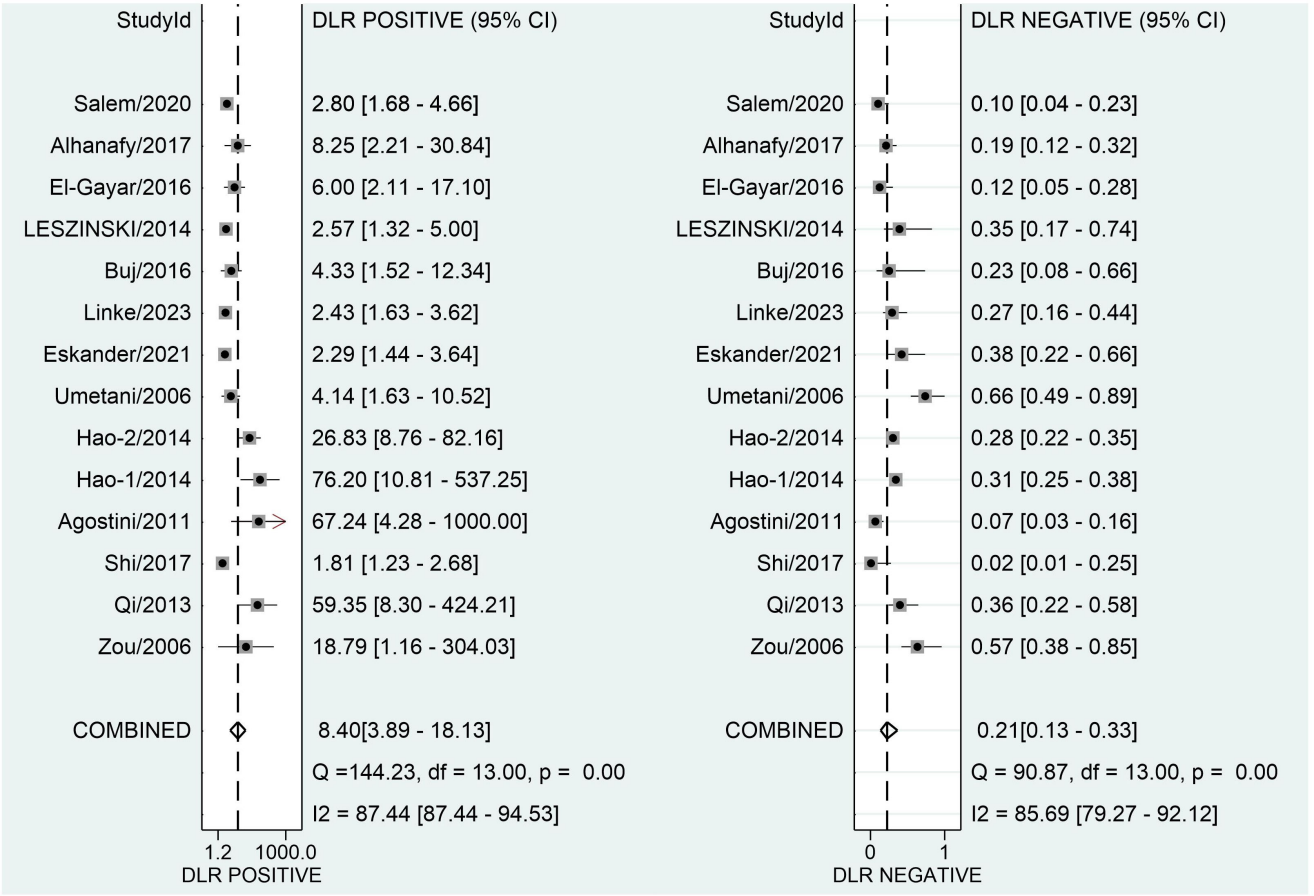
Forest plot of the PLR and NLR of ALU-based cfDNA in the diagnosis of CRC.

**Figure 3** depicts forest plots and aggregated sensitivity and specificity estimates for all relevant studies. The studies were diverse in terms of sensitivity (I2 = 87.37%) and specificity (I2 = 91.29%). The combined sensitivity and specificity were 0.81 (95% CI = [0.70, 0.89]) and 0.90 (95% CI = [0.79, 0.96], respectively. **Figure 4** shows the predicted combined DOR of 40.48 (95% CI = [17.87, 92.19]). **Figure 5** shows the SROC curve and its related 95% CI and prediction outlines, with an estimated overall AUC of 0.92 (95% CI = [0.89, 0.94]), showing that the test has adequate diagnostic accuracy. **Figure 6** depicts forest plots and combined values of PLR and NLR, which are thought to be more relevant than sensitivity and specificity in clinical settings. The pooled estimate of PLR= 8.40 (95% CI = [3.89, 18.13]) shows that the likelihood of positive test findings in CRC patients is nearly eight times that of people without CRC. Furthermore, the pooled NLR = 0.21 (95% CI = [0.13, 0.33]) indicates that the likelihood of receiving negative test findings in patients without CRC is about five times that of those with CRC.

### Threshold effect

The threshold effect is hypothesized to result from changes in sensitivity and specificity. In this work, we investigated the threshold impact using Spearman’s correlation coefficient.

### Sensitivity analysis and publication bias

**Figure 7:**
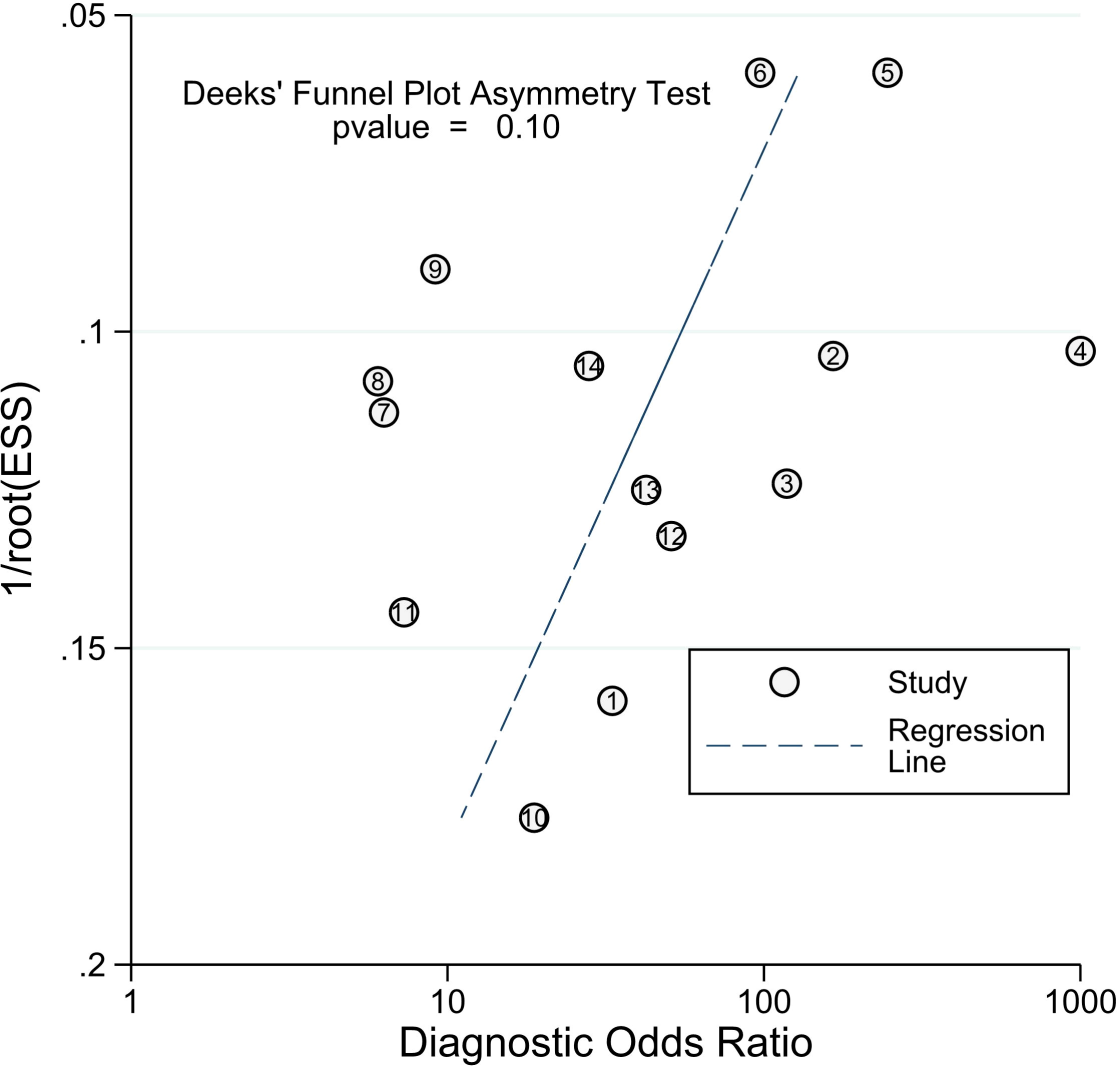
Deeks’ funnel plots for the assessment of potential bias in the meta-analysis for diagnostic value of ALU-based cfDNA in CRC.

Our study revealed a combined sensitivity of 0.81 (95% CI = [0.70, 0.89]) and specificity of 0.90 (95% CI = [0.79, 0.96]) for ALU-based cfDNA diagnostic accuracy in CRC. This sensitivity (0.81) indicates that ALU-based cfDNA testing correctly identifies a significant proportion of true positive cases (CRC patients). This is crucial for early detection and minimizing false negatives. In addition, This specificity (0.90) implies that ALU-based cfDNA testing has a low rate of false positives, reducing unnecessary anxiety and follow-up procedures for healthy individuals. Furthermore, To examine publication bias, we employed Deeks’ funnel plot. The funnel plots did not exhibit symmetry (see **Figure 7**). The Deeks funnel plot test for the diagnostic value of ALU-based cfDNA yielded a p-value of 0.10.

### Prognostic meta-analysis

#### Study characteristics and quality assessment

A total of 942 participants from 4 studies were analyzed, comprising 808 individuals with CRC and 134 healthy controls. All eligible studies were retrospective and involved patients from four different countries. In the prognostic analysis, samples were categorized as serum (n = 1) (55), plasma (n = 1) (56), FFPE (n = 1) (57), and tissue (n = 1) (58). In terms of prognostic value, one study analyzed ALU83/244 (56), another studied ALU115/247 (55), and two others explored ALU methylation (57, 58). Two studies used ALU analysis by q-PCR (55, 56),, one utilized tissue microarrays (57), and another employed bisulfite-PCR and pyrosequencing (58). The meta-analysis included studies from Korea (58), the Netherlands (57), Italy (56), and Germany (55). Concerning different stages of CRC, one study included only stage IV (55), one encompassed all stages (58), one focused on stages I/II (57), and another did not specify the stages of CRC included (56). Further details about the included studies can be found in **Table 2**.

**Table 2:** Characteristics of the included studies for the prognostic value of ALU in CRC patients.

NO.	First Author	Country	ALU-based cfDNA type	Specimen	Tumor stages	Detection Method	No. of P	No. of C	HR (95%CI)	Clinical evidence	Follow-up (Months)	Ref
1	Ye-Young Rhee (2012)	Korea	ALU methylation	Tissue	I-IV	bisulfite-PCR and pyrosequencing	207	–	2.551(0.858–7.343)	OS	80	(58)
2	A Benard (2013)	Netherlands	ALU methylation	FFPE	I/II	tissue microarrays	219	78	1.269(0.681–2.361)	OS	150	(57)
3	Chiara Bedin (2016)	Italy	ALU83	plasma	–	Bisulfite modification and methylation-specific real-time PCR	114	56	3.49(1.58–7.71)	OS	150	(56)
4	Chiara Bedin (2016)	Italy	ALU244	plasma	–	Bisulfite modification and methylation-specific real-time PCR	114	56	2.7 (1.25– 5.84)	OS	150	(56)
5	Isabel Anzinger (2023)	Germany	ALU115	Serum	IV	qPCR	268	–	2.9(1.8–4.8)	OS	120	(55)
6	Isabel Anzinger (2023)	Germany	ALU247	Serum	IV	qPCR	268	–	2.2(1.3–3.6)	OS	120	(55)

### Correlation between ALU level and OS

**Figure 8:**
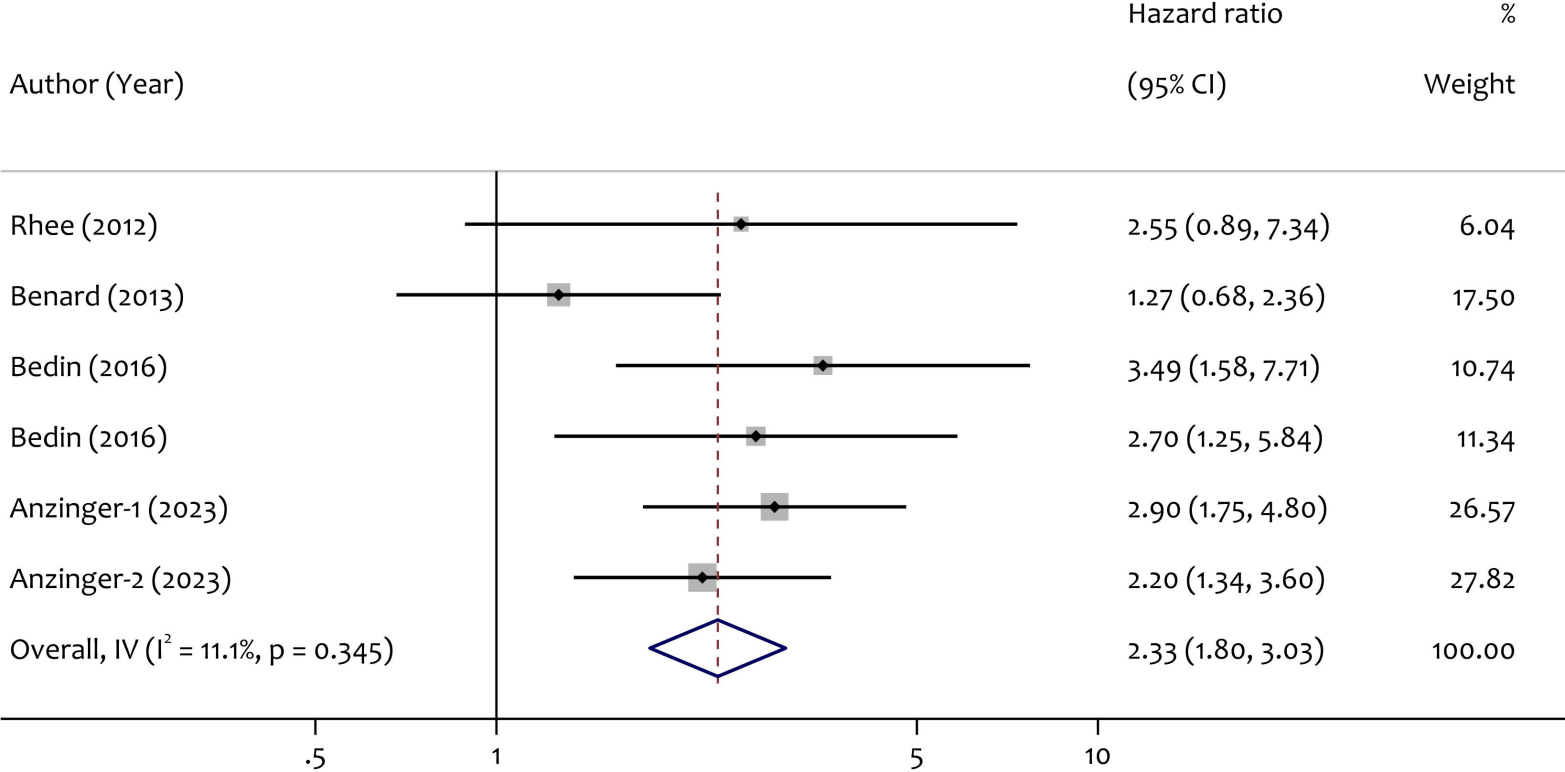
Forest plot of the hazard ratios of ALU-based cfDNA level in CRC prognosis.

Because of the variability among studies measuring OS (I2 = 11.1%), a fixed model was chosen. HR= 2.33 (95% CI= [1.80, 3.03]) suggests that greater ALU-based cfDNA levels are related with a shorter OS in CRC (**Figure 8**).

### Publication bias

**Figure 9:**
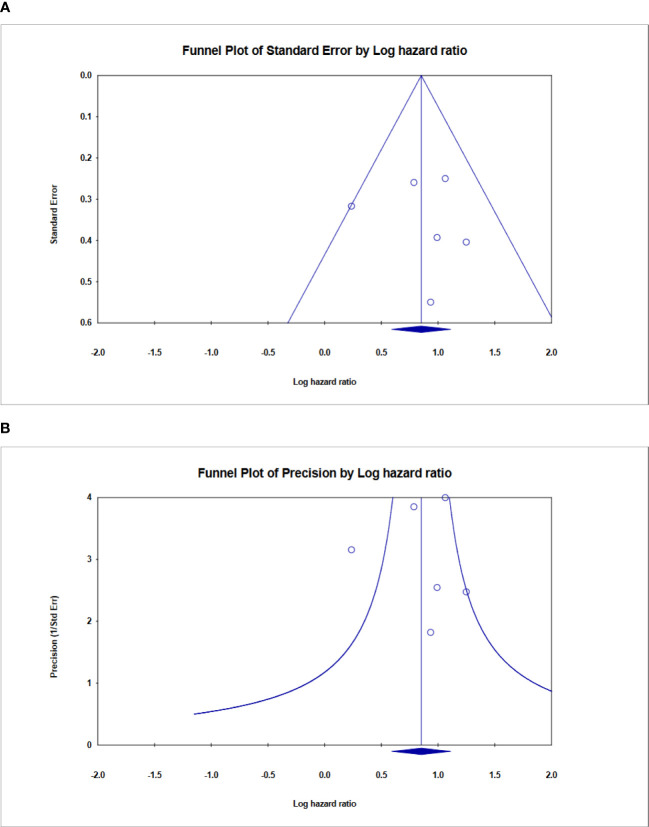
Funnel plot showing no publication bias in terms of prognostic odds ratio significance among the included. **(A)** Funnel plot of standard error, **(B)** Funnel plot of Precision for ALU-based cfDNA in CRC.

Finally, Begg’s funnel plot was utilized to assess publication bias (**Figure 9**). There was no evident publication bias in the quantitative synthesis for evaluating OS. However, due to the small number of included papers, publication bias cannot be completely excluded out.

## Discussion

In recent years, LB has become increasingly common for cancer diagnosis. To determine the potential diagnostic and prognostic impact of ALU-based cfDNA, 17 papers were analyzed to identify ALU-based cfDNA sequences as a cancer biomarker. In this exploratory meta-analysis of 13 trials using 14 sets of data, the pooled analysis had a diagnostic sensitivity of 0.81 and a specificity of 0.90. These two typical characteristics verified the accuracy of ALU-based cfDNA as a viable noninvasive predictor for CRC detection.

The advent of Next-Generation Sequencing (NGS) has revolutionized the landscape of genomic analysis, offering a comprehensive tool that surpasses the capabilities of traditional qPCR systems, particularly in the context of ALU sequence coverage. NGS provides a hypothesis-free approach that does not require prior knowledge of sequence information, enabling the discovery of novel genes and the quantification of rare variants and transcripts with higher sensitivity (59). NGS has transformed the LB field for CRC through its comprehensive analysis capabilities. The ability of NGS to extensively map ALU sequences within cfDNA is vital for uncovering an array of biomarkers, offering a deeper understanding of CRC’s genetic intricacies. This enhanced level of detail is fundamental for the precise diagnosis and the prognostic evaluation of CRC. By encompassing a broader spectrum of ALU sequences, NGS ensures a more accurate and dependable measure of cfDNA’s biomarker potential, which may significantly improve the clinical management and outcomes for CRC patients (33, 60).

A recent meta-analysis on the use of circulating cfDNA as a diagnostic marker for CRC indicated that while the sensitivity of circulating cfDNA is unsatisfactory, its specificity remains acceptable for CRC diagnosis (61). Furthermore, another systematic review revealed elevated concentrations of ALU repetitive elements in cancer patients, whereas these concentrations were reduced in control groups, benign conditions, various cancer stages, and other diseases. The total ALU (115 and 247) sequence levels emerge as potential biomarkers for investigative purposes and cancer prognosis (32).

Our study’s pooled DOR of 40.58 indicated a good overall accuracy. Furthermore, the PLR was found to be 8.40, whereas the NLR was discovered to be 0.21. These data show that the meta-analysis’s good likelihood ratios may indicate the results’ robustness and accuracy. Furthermore, ROC is often used to define overall test performance, and AUC acts as a measuring indication; the AUC of SROC for ALU-based cfDNA was 0.90, showing that ALU-based cfDNA has a reasonably good accuracy for CRC detection. Heterogeneity is an important issue in meta-analysis. The I^2^ test revealed high heterogeneity in our study’s trials. The threshold effect is typically the predominant source of heterogeneity in diagnostic meta-analyses. To evaluate the likely cause of variability, we used meta-regression to examine the features of included studies, including publication year, research location, kind of specimens, detection techniques, measuring objects, number of cases, and four major domains in QUADAS-2.

Finally, our analysis revealed that study quality played a significant role in the substantial heterogeneity, implying that the study design with high-risk biases of “Patient selection,” “Index Test,” “Reference Standard,” and “Flow and Timing” may be more likely than other characteristics to affect diagnostic accuracy. Heterogeneity may have increased due to other factors such as age, tumor type, metastasis, TNM staging, surgery method, and treatment regimen, which were not investigated in the current investigation due to a lack of data availability. Although publication bias can be a concern in meta-analyses, Deeks’ funnel plot asymmetry test found no such bias, indicating that our meta-analysis results for diagnosis and prognosis are credible. Furthermore, blood-based (plasma or serum) tests showed considerably better overall diagnostic accuracy and were created as a non-invasive diagnostic biomarker for CRC. On the other hand, the meta-analyses revealed that ALU-based cfDNA was a potential biomarker for predicting OS in CRC patients. In our prognostic analysis, which comprised four trials and six data sets, the pooled results showed that patients with low levels of ALU-based cfDNA had a 2.33 (HR = 2.33) times reduced chance of poor OS. Furthermore, there was substantial variability across the prognostic meta-analyses. A significant range in sample types was seen throughout the studies that contributed to the predictive results. However, several issues still limit its implementation in clinical practice. There is no consensus on the utility of cfDNA testing in cancer patients. We remain skeptical of any future suggestions for ALU-based cfDNA. A future study will assist in determining this. CEA and CA19–9 are commonly utilized clinical indicators for the diagnosis and prognosis of CRC. Higher CEA concentrations occur in only 5%-40% of CRC patients, while positive findings are frequently reported in cancer-free patients who suffer from benign conditions such as liver injury or inflammatory disorders (53, 62). As a result, we hope and aim to demonstrate that ALU-based cfDNA can be a therapeutically valuable surrogate marker.

Our study has several important strengths. First, this is the first study to comprehensively examine the association between the amount of ALU-based cfDNA and its importance in diagnosing and predicting outcomes in CRC patients. Both diagnostic and prognostic meta-analyses were performed based on a sufficient number of publications. Second, our meta-analysis has uncovered interesting results, paving the way for future research. However, there were also several limitations in our work. Firstly, all publications regarding the prognosis of ALU-based cfDNA have exclusively focused on evaluating OS rather than other endpoints such as DFS, PFS, and CSS. In addition, This meta-analysis was limited to the evaluation of univariate OS due to the available studies that could be included in the analysis and highlight the necessity for further investigations into the prognostic impact of ALU-based cfDNA using other time-to-event (TTE) end-points. Furthermore, little research has been conducted to determine the prognostic value of ALU-based cfDNA in CRC. Second, our research relied on data from published studies rather than individual patient data (IPD), which limited the ability to conduct a uniform analysis of all data. So, Obtaining raw data is crucial for survival prediction and analysis. Moreover, we did not extend the search to non-English publications, which may have resulted in bias because good results are more easily accepted by English-language journals. Even though progress has been made in spotting and predicting CRC, we still urgently need better markers to improve early detection and predict patient outcomes more accurately (63). Future research directions for ALU-based cfDNA in CRC include validation across diverse populations, longitudinal studies, integration with other biomarkers, standardization of detection techniques, clinical utility assessment, exploration of tumor-specific ALU-based cfDNA, prospective cohort studies, patient acceptance evaluation, cost-effectiveness analysis, and collaboration for consensus guidelines. These efforts will refine ALU-based cfDNA’s clinical applicability and address limitations.

## Conclusion

The findings of this systematic review and meta-analysis have important implications for the clinical management of CRC. The results indicate that serum ALU-based cell-free DNA (cfDNA) is a promising biomarker that can aid in the diagnosis and prognosis of CRC. This non-invasive test could potentially be incorporated into routine clinical practice to supplement existing diagnostic tools and provide valuable prognostic information to guide treatment decisions.

The next steps should involve further large-scale, high-quality studies to validate the diagnostic and prognostic performance of ALU-based cfDNA testing in diverse patient populations. Additionally, research is needed to optimize the analytical methods and establish standardized protocols for the measurement of ALU-based cfDNA. Once these steps are taken, ALU-based cfDNA testing could become a valuable addition to the clinical armamentarium for managing patients with CRC, potentially leading to earlier diagnosis, more personalized treatment, and improved patient outcomes.

## Data availability statement

The original contributions presented in the study are included in the article/**Supplementary Material**. Further inquiries can be directed to the corresponding authors.

## Author contributions

MT: Data curation, Investigation, Writing – original draft, Writing – review & editing. LA: Conceptualization, Data curation, Project administration, Supervision, Validation, Visualization, Writing – review & editing. SM: Conceptualization, Data curation, Project administration, Supervision, Validation, Visualization, Writing – review & editing. AK: Conceptualization, Data curation, Project administration, Supervision, Validation, Visualization, Writing – review & editing. TJ: Conceptualization, Data curation, Formal analysis, Methodology, Project administration, Software, Supervision, Validation, Visualization, Writing – review & editing.
